# Validation of the modified Microlife blood pressure monitor in patients with paroxysmal atrial fibrillation

**DOI:** 10.1007/s00392-019-01567-y

**Published:** 2019-11-07

**Authors:** Nina Huppertz, Gregory Y. H. Lip, Deirdre A. Lane

**Affiliations:** 1 Sandwell and West Birmingham Hospitals NHS Trust, Birmingham, UK; 2 Liverpool Centre for Cardiovascular Science, Institute of Ageing and Chronic Disease, University of Liverpool and Liverpool Heart and Chest Hospital, William Henry Duncan Building, 6 West Derby Street, Liverpool, L7 8TX UK; 3 Aalborg Thrombosis Research Unit, Department of Clinical Medicine, Aalborg University, Aalborg, Denmark

**Keywords:** Atrial fibrillation, Blood pressure, Detection, Sensitivity, Specificity

## Abstract

**Aims:**

Undiagnosed atrial fibrillation (AF) accounts for 6% of all strokes, therefore early detection and treatment of the arrhythmia are paramount. Previous research has illustrated that the Microlife WatchBPO3 AFIB, an automated blood pressure (BP) monitor with an inbuilt AF algorithm, accurately detects permanent AF. Currently, limited data exist on whether the modified BP monitor is able to detect paroxysmal AF (PAF). Therefore, this study aims to assess the accuracy of the Microlife WatchBPO3 AFIB monitor to detect PAF against a pacemaker reference standard over a 24-h period.

**Methods and results:**

Forty-eight patients with a pacemaker implanted for sick sinus syndrome and previously documented fast AF participated. Sensitivity of the atrial pacemaker lead was set to allow detection of signals of ≥ 0.5 mV. Patients engaged in their normal daily routine whilst wearing the modified BP monitor. The modified BP monitor demonstrated an overall sensitivity of 76.0% and specificity of 80.8% for detecting PAF. This sensitivity and specificity increased to 100% and 83.1%, respectively, for patients that achieved more than 80% successful BP readings. Compared to day-time readings, night-time readings also demonstrated a lower proportion of movement artefact (14.4% vs. 3.4%), and therefore, a higher sensitivity and specificity of 100% and 84.9%, respectively, for detecting PAF.

**Conclusion:**

The Microlife WatchBPO3 AFIB device has an acceptable diagnostic accuracy to detect PAF; however, movement artefact affects the accuracy of the readings. This modified BP monitor may potentially be useful as a screening tool for AF in patients at high risk of developing stroke.

**Electronic supplementary material:**

The online version of this article (10.1007/s00392-019-01567-y) contains supplementary material, which is available to authorized users.

## Introduction

Atrial fibrillation (AF) is the most commonly encountered arrhythmia in clinical practice and is a powerful independent risk factor for stroke [1]. It is estimated that 15% of all strokes is attributable to AF, the majority of which are more severe, more likely to lead to disability, and more often fatal than other forms of strokes [2]. Since undiagnosed AF accounts for 3.8% to 6.1% of all strokes, detection of the arrhythmia is extremely crucial [3]. In spite of guidelines suggesting opportunistic manual pulse palpation in patients over 65 years old, symptomatic, or at high risk for stroke, paroxysmal AF (PAF) is frequently able to evade routine detection due to its intermittent, brief, and usually asymptomatic episodes [4].

Previous research has shown that the Microlife WatchBPO3 AFIB, a modified automated blood pressure (BP) monitor, is able to accurately detect permanent AF with a sensitivity of 94.9–95% and specificity of 86–89.7% in outpatient clinics and primary care centres [5, 6]. With the exception of one study that demonstrated a sensitivity of 100% and specificity of 93% to detect transient AF over 30 days with multiple successive readings, there is currently a lack of research investigating the performance of the Microlife WatchBPO3 AFIB over extended periods in at-home settings, and so it is generally unknown if this monitor is able to detect PAF with the same level of accuracy [5].

Therefore, the purpose of this study was to examine the diagnostic accuracy of this modified BP monitor to detect PAF over a 24-h period among patients with a pacemaker implanted for sick sinus syndrome, who had previously documented fast AF and atrial high-rate episodes (AHRE). Glotzer et al. [7] confirmed that pacemakers have the ability to document brief episodes of AF using atrial high-rate episodes (AHRE) with an extremely high sensitivity of 100% and specificity of 97.6%. Given the high level of precision with which pacemakers can document brief episodes of AF, comparing the accuracy of the modified BP monitor against this, could provide valuable information for healthcare practice. If successful in identifying PAF, the Microlife WatchBPO3 AFIB may be useful as a screening tool in patients who are at high risk of developing stroke or are referred for ambulatory BP tests.

## Methods

This observational study compared the diagnostic accuracy of the Microlife WatchBPO3 AFIB (Microlife, Heerbrugg, Switzerland) to detect PAF against the reference standard of an implanted pacemaker. The study protocol was approved by the West Midlands-Black Country Research Ethics Committee of the Health Research Authority (REC 13/WM/0382, IRAS 114264), and the Sandwell and West Birmingham Hospitals NHS Trust Research and Development Department (R&D 13CARD56). The study was registered with the National Institute of Health Clinical Trials Registry (Registration No. NCT02442505). All participants provided written informed consent.

### Setting and participants

Patients were recruited from the cardiology outpatient clinic register at one NHS Trust in the West Midlands, UK. Patients with an implanted pacemaker for sick sinus syndrome and previously documented AF or AHRE were invited to participate in the study. Exclusion criteria included patients that (i) were unable to provide written informed consent, (ii) had previous pacemaker sensing issues on either the atrial or ventricular pacemaker lead, (iii) diagnosed with permanent AF, or (iv) had a VVI pacemaker.

### Test procedure

Patients attended the cardiology outpatient department on two consecutive days. Subsequent to obtaining written informed consent, details on their demography, medical history, and pacemaker were recorded. Patients had their pacemaker interrogated, during which the sensitivity on the atrial lead was temporarily altered to allow for detection of signals of at least ≥ 0.5 mV and rates of at least ≥ 180 bpm. If the atrial lead possessed an autosensing feature, this was disabled. No other settings were changed. Patients were then fitted with the Microlife WatchBPO3 AFIB monitor. Based on their upper arm circumference, an appropriate cuff size was chosen and fitted 2–3 cm above the elbow. The cuff tube was placed around the upper arm, pointed upward, and mounted over the patient’s shoulder to allow the monitor to be placed in a pouch that hung around the neck. To mimic current clinical practice for 24-h ambulatory BP monitoring and therefore allow for a better attempt at generalising results to the real-world setting, the modified BP monitor was automatically timed to take a reading every 30 min from 08:00 to 22:00 and every 60 min from 22:00 to 08:00 [8]. Patients were asked to relax their arm, remain still, and refrain from talking during the BP measurement. Prior to leaving, the monitor was switched to ‘ambulatory’ mode. If patients were symptomatic over the 24 h, they were encouraged to record their symptoms on a diary sheet. Patients were then asked to engage in their usual daily routine for the next 24 h out of hospital. On the following day, patients returned to the department to have their modified BP monitor removed and the data were downloaded from the device via computer software. Their pacemaker was interrogated to assess for AF or AHRE occurring in the last 24 h and thereafter re-programmed to the original settings. Pacemaker-detected episodes of AF were analysed by a cardiac physiologist to confirm their accuracy.

### Detection methods

Microlife WatchBPO3 AFIB is an oscillometric BP monitor that possesses an additional independent feature to detect AF. The inbuilt algorithm analyses pulse irregularity during the last 10 beats of cuff deflation by calculating the irregularity index, which is defined as the “standard deviation of the time intervals between successive heartbeats divided by the mean of the intervals for the total number of beats analysed” [9]. To exclude incidental arrhythmias, each interval that deviates by 25% from the mean is discarded from the analysis. Diagnosis of AF is made if the irregularity index is above the threshold value of > 0.06 [9]. On the other hand, pacemaker detection of an atrial tachyarrhythmia is a continuous process. Most dual-chamber pacemakers employ an automatic mode switch (AMS) algorithm to detect atrial tachyarrhythmias, which was designed to prevent its tracking through to the ventricles and thereby maintain atrioventricular synchrony. By doing so, it also triggers an electrogram (EGM) storage of an AHRE [10]. AMS proves a reliable marker for detection of atrial tachyarrhythmias with a reported sensitivity and specificity of 98.1% and 100%, respectively [11]. Furthermore, Microlife WatchBPO3 AFIB is also able to detect movement artefact, weak signals, and cuff leakage, providing a readout with a ‘remark’ for each individual reading.

### Outcomes

The primary outcome of this study is to determine the sensitivity, specificity, positive predictive value (PPV), and negative predictive value (NPV) of the modified BP monitor to detect PAF. One large clinical hypertension trial suggested that the percentage of valid BP readings obtained during 24-h should exceed 80% of all readings attempted. Therefore, the diagnostic accuracy (sensitivity, specificity, PPV, and NPV) of the modified BP monitor was also compared between patients that achieved above and below this threshold [12]. Subgroup analyses of the diagnostic accuracy of the modified BP monitor during any movement artefact were also performed by assessing day- and night-time readings separately. Night-time was determined by each patients’ stated waking and sleeping hours on their diary sheet. For patients that did not provide this information, a set standard of 22:00–08:00 was used.

### Statistical analyses

The Kolmogorov–Smirnov test was used to determine the normality of the distribution for continuous variables. Normally distributed variables are portrayed by mean ± standard deviation (SD), while non-normally distributed variables are reported as median and interquartile range (IQR). Sensitivity, specificity, PPV, and NPV were calculated as simple proportions with corresponding 95% confidence intervals (95% CI). 95% CI were computed based on the binomial approximation. Pearson’s *χ*^2^ test was used to determine differences between categorical variables. For categorical variables with an expected count of less than 5, the likelihood ratio *χ*^2^ test was used instead. Pearson’s correlation test was used to determine univariate correlations between the percentage of AF readings detected by the modified BP monitor and the daily maximum burden of AF detected by the pacemaker. Statistical analysis was performed using IBM SPSS 24 (IBM Corporation). Statistical significance was set at *p* < 0.05.

## Results

### Clinical characteristics

From 178 potential candidates for this study, 121 (68%) declined to participate. Fifty-seven patients met the inclusion criteria. Six withdrew, two were newly diagnosed with permanent AF, and one patient was deemed unsuitable as data between the implanted pacemaker and modified BP monitor could not be accurately compared, resulting in a total of 48 (84%) patients being included in the final analysis. These patients ranged in age from 44 to 92 years with a mean (SD) age of 71.6 (10.9) years. The majority were male (58.3%), of white ethnicity (70.8%), had diagnosed hypertension (66.7%), and were receiving anticoagulation therapy for paroxysmal AF (60.4%). Both clinical characteristics of and medications taken by included patients are summarised in Table 1.

**Table 1:** Clinical characteristics, medications, and pacemaker details of included patients

Variable	Included patients (*n* = 48)
Current age (years)	71.6 ± 10.9
Age at pacemaker implant (years)	67.9 ± 11.0
Gender (*n*, %)	
Male	28 (58.3)
Female	20 (41.7)
Height (cm)	167.9 ± 9.7
Weight (kg)	76.1 [21.0]
Body mass index (kg/m^2^)	27.0 [8.4]
Ethnicity (*n*, %)	
White	34 (70.8)
Asian (Indian, Pakistani, Bangladeshi)	10 (20.8)
Black/Caribbean	2 (4.2)
Mixed	2 (4.2)
Congestive HF (*n*, %)	1 (2.1)
Hypertension (*n*, %)	32 (66.7)
Diabetes mellitus (*n*, %)	
Type I	0 (0)
Type II	14 (29.2)
Previous stroke (*n*, %)	
Ischaemic	3 (6.3)
Haemorrhagic	2 (4.2)
Unspecified	3 (6.3)
Vascular disease (*n*, %)	
Prior myocardial infarction	5 (10.4)
Coronary artery disease	12 (25.0)
Peripheral artery disease	0 (0)
Hyperlipidaemia (*n*, %)	19 (39.6)
Other cardiovascular disease (*n*, %)	
Prior cardiac surgery	2 (4.2)
Congenital	0 (0)
Valvular	1 (2.1)
Pericarditis	0 (0)
Cardiomyopathy	0 (0)
Medications (*n*, %)	
Anticoagulants	29 (60.4)
Antiplatelets	16 (33.3)
ACE-inhibitors	15 (31.3)
Angiotensin receptor blocker	8 (16.7)
Beta-blockers	23 (47.9)
Calcium-channel blockers	12 (25.0)
Digitalis	2 (4.2)
Diuretics	17 (35.4)
Statins	31 (64.6)
Vasodilators	8 (16.7)
Device model (*n*, %)	
Medtronic Adapta	8 (16.7)
Medtronic Advisa	14 (29.2)
Medtronic Ensura	6 (12.5)
Medtronic Sensia	8 (16.7)
St. Jude Accent	1 (2.1)
St. Jude Assurity	7 (14.6)
St. Jude Azure	2 (4.2)
Sorin Reply	2 (4.2)
Mode (*n*, %)	
DDD	3 (6.3)
DDDR	16 (33.3)
DDI	1 (2.1)
AAI-DDD	10 (20.8)
AAIR-DDDR	17 (35.4)
AAIR	1 (2.1)
Indication for implant (*n*, %)	
Sinus arrest	19 (39.6)
Sinus bradycardia	18 (37.5)
Brady-tachy syndrome	6 (12.5)
Chronotropic incompetence	1 (2.1)
Unspecified SSS	4 (8.3)

Mean ± standard deviation given for normally distributed variables, median [interquartile range] given for not normally distributed variables. Number (%) given for categorical variables

### Pacemaker function

Patients participating in the study had the following pacemakers in situ: Medtronic (75.1%), St. Jude Medical (20.6%), and Sorin (4.2%). Most pacemakers were dual-chamber (97.9%) and implanted for symptomatic sinus arrest (39.6%). Further pacemaker details are provided in Table 1. Patients displayed normal pacemaker function over the 24 h testing period.

### Detection of paroxysmal AF

**Fig. 1:**
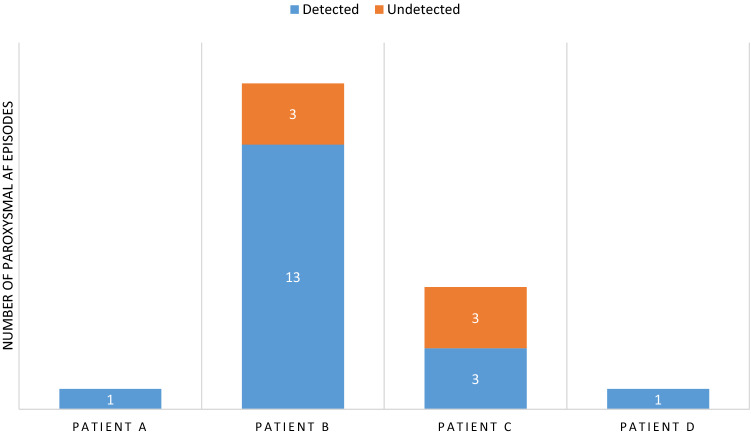
Distribution of true paroxysmal AF episodes with detection ability of Microlife WatchBPO3 AFIB

From the 48 patients, 1595 modified BP monitor readings were collected for individual analysis. From these recordings, the prevalence of AF was 1.6% with a maximum AF burden of 5.4%, over the 24-h testing period. Overall, 4 out of 48 patients (8.3%) collectively exhibited 24 true PAF episodes. In these patients, 30-day AF burden ranged from 0.6 to 77.6%, with those that had the highest number of AF episodes also having the highest AF burden. Figure 1 highlights the distribution of these episodes, showcasing those that were detected or undetected by the Microlife WatchBPO3 AFIB monitor. Out of the 48 patients, only a single individual (Patient B) reported one episode of light-headedness that corresponded to a true AF event. From the 1595 modified BP monitor readings, 19 were true positives, 9 were false negatives, 300 were false positives, and 1270 were true negatives (Table 2). Compared to the pacemaker, the Microlife WatchBPO3 AFIB monitor demonstrated a sensitivity of 76.0%, specificity of 80.8%, PPV of 5.9% and a NPV of 99.5% to detect PAF. In the cohort that achieved more than 80% successful BP readings, the sensitivity and specificity increased to 100% and 82.3%, respectively. Patients that did not achieve this threshold of successful BP readings demonstrated a lower sensitivity of 76.0% and specificity of 76.9%. Due to only one true positive occurrence in this cohort, PPV was low at 0.5%, whilst NPV was high at 100%.

**Table 2:** Modified BP monitor diagnoses of paroxysmal AF vs. pacemaker diagnoses of paroxysmal AF

	Positive AF pacemaker diagnosis	Negative AF pacemaker diagnosis
Positive AF modified BP monitor diagnosis	19	300
Negative AF modified BP monitor diagnosis	6	1270

### Effect of movement artefact on diagnostic accuracy

Overall, there were more day-time readings than night-time readings (73.9% vs. 26.1%). Day-time readings demonstrated more movement artefact than night-time readings but the difference was not significant (14.4% vs. 3.4%, likelihood ratio = 3.2, *p* = 0.08). Compared to night-time readings, the modified BP monitor demonstrated lower sensitivity, specificity, PPV, and NPV during day-time readings. Table 3 summarises the diagnostic accuracy of the Microlife WatchBPO3 AFIB monitor to detect PAF in each setting.

**Table 3:** Sensitivity, specificity, PPV, and NPV with 95% CI of the Microlife WatchBPO3 AFIB to detect paroxysmal AF in various settings

	Sensitivity (%)	Specificity (%)	PPV (%)	NPV (%)
Overall (24-h) AF detection	76.0 (74–78)	80.8 (79–83)	5.9 (5–7)	99.5 (99–100)
Day-time AF detection	68.4 (66–71)	79.3 (77–81)	5.1 (4–6)	99.4 (99–100)
Night-time AF detection	100 (99^a^–100)	84.9 (83–87)	8.8 (7–10)	100 (99^a^–100)
AF detection in < 80% successful BP readings	76.0 (59–96)	76.9 (70–79)	15.4 (8–21)	98.3 (97–100)
AF detection in > 80% successful BP readings	100 (99^a^-100)	83.1 (80–86)	0.5 (0–4)	100 (99^a^–100)

^a^One-sided 97.5% confidence interval

### Correlation between AF burden on pacemaker and AF detected by modified BP monitor

A strong positive correlation was found between the percentage of AF burden (i.e. amount of time a patient spent in AF) recorded by the pacemaker over 30-days and the percentage of readings positive for AF detected by the modified BP monitor during the 24-h ambulatory period for all patients (*r* = 0.70, *p* < 0.001) (Supplemental Fig. 1). This correlation was further strengthened in patients that had more than 30% of readings positive for AF (*r* = 0.89, *p* = 0.001) highlighting that true-positive readings of AF are more likely to be detected by the modified BP monitor in patients with a higher overall burden of AF.

## Discussion

This is the first study to investigate the diagnostic accuracy of the Microlife WatchBPO3 AFIB to detect PAF over a 24-h ambulatory period. Our findings show that the Microlife WatchBPO3 AFIB device has an acceptable diagnostic accuracy to detect PAF; however, movement artefact affects the accuracy of the readings.

Compared to previous research that has established high diagnostic accuracy of modified BP monitors to detect permanent AF in outpatient clinics, the overall sensitivity and specificity reported in the current study was marginally lower [5, 6, 13]. Given that PAF is more difficult to detect in light of its brief and transient episodes, it is unsurprising that the sensitivity and specificity is higher in a permanent AF cohort. Ensuring the modified BP monitor has a high sensitivity is important for clinic visits, as screening opportunities are restricted to a limited number. This does not necessarily apply to ambulatory BP monitoring, which can collect up to 38 measurements a day, allowing for a higher chance of overall detection. Therefore, a lower sensitivity may be acceptable for frequent readings performed over an extended period of time.

Only one other study has attempted to detect PAF over 30-days at home by performing a maximum of three successive readings on a daily basis [5]. Despite the high diagnostic accuracy reported, self-performing sequential readings relies heavily on patient compliance for its efficacy. It may be a useful tool for detection of PAF in patients that already possess modified BP monitors at home, but issuing these for the general population to use over a prolonged period as part of a screening process is time-consuming and unlikely to be cost-effective. Only one daily set of readings may fail to detect transient episodes, and therefore, 24-h ambulatory monitoring is a better alternative to increase the chances of detecting new AF cases.

Of note, the sensitivity and specificity in our cohort increased to 100% and 83.1% respectively, when only considering patients who achieved more than 80% of successful BP readings. It is important to note that even when a BP reading is not provided, the Microlife WatchBPO3 AFIB monitor has the ability to determine whether AF was occurring at the time of inflation due to the independently functioning algorithm for detection. Despite this, proposed standards suggest that a satisfactory recording should consist of at least 70–80% successful BP readings, and therefore, this criterion should be applied if using ambulatory modified BP monitors for the detection of PAF [14, 15]. One drawback of this modified BP monitor to detect PAF is the false positive rate of 19.2%, suggesting that approximately 1 in 5 readings was incorrectly identified as AF. Compared to patients who had a successful ambulatory recording, patients that did not display a higher false positive rate (34.2% vs. 16.9%), further strengthening the argument that a successful ambulatory recording is required to improve the diagnostic accuracy for the detection of PAF. This study could not investigate the proportion of false positive readings potentially due to other arrhythmias. Despite the pacemaker’s continuous monitoring for abnormal rhythms, it does not record an EGM for individual premature atrial or ventricular ectopics. Since patients with pacemakers are more likely to have cardiac arrhythmias, a large percentage of false-positive readings can be anticipated. Wiesel et al. [16] illustrated that 62% of ventricular ectopics and 43% of atrial ectopics were accurately identified by the modified BP monitor as not being AF, resulting in a low specificity of 50% for patients with frequent ectopics. However, restricting this tool to patients without recurrent ectopics would result in at least half of this cohort not receiving its benefit. No formal indications exist on how false-positive readings may affect the diagnostic accuracy if screening patients at high risk for stroke over a 24-h ambulatory period.

Furthermore, false-positive readings may arise as a result of excessive movement during measurements. Unlike in clinic visits, movement artefact is more likely to occur with prolonged ambulatory recording. Compared to night-time readings, our findings demonstrated that day-time readings had a higher proportion of movement artefact, which potentially explains the lower overall diagnostic accuracy and contribution to the increased false-positive rate by 7%. Because the modified BP monitor relies on the brachial pulse to determine irregularity, any excessive movement that would obstruct its analysis can cause erroneous results. Ensuring patients optimise their positioning during the measurements should be emphasized in all ambulatory BP recordings. Future research should compare the modified BP monitor to a 24-h ECG, which would provide more insight on the proportion of false-positive readings discernable to either movement or other arrhythmias.

Given the high false-positive rate, criteria are required to determine which patients receive additional ECG monitoring to officially diagnose PAF. Based on the correlation between false-positive readings and ectopics, Kollias et al*.* [17] concluded that patients over 50 years old with > 30% of positive readings are highly likely to have true AF. One trial revealed more than 5.5 h of AHRE was shown to significantly increase the risk of stroke [18]. If a patient had > 30% of all true-positive readings for AF, it would suggest that PAF was present for at least 7 of the 24 h. By prioritising further testing to patients with > 30% of positive readings, the economic burden created by false-positive readings could be reduced. The current study provides further insights, highlighting a strong positive correlation between the overall time spent in AF over 30-days as detected by the pacemaker and the proportion of true-positive readings for AF over the 24-h ambulatory period as detected by the modified BP monitor. Patients with > 30% positive readings for AF had a significantly higher overall burden of AF over 30-days, suggesting that prioritising follow-on diagnostic testing to this group ensures that patients with a significant amount of PAF can be identified and administered appropriate treatment to decrease the risk of stroke. By employing this criteria in clinical practice, the Microlife WatchBPO3 AFIB monitor could function as an accurate triage tool in patients with hypertension, in those at high risk of developing AF (≥ 65 years with > 1 stroke risk factor), and in those at high risk of stroke.

It is important to note that PAF can never truly be ruled-out. Due to its nature, brief episodes are common and can occur in between the automatically timed BP readings or outside of the 24-h monitoring period altogether. Therefore, clinicians should not rely solely on the modified BP monitor to draw any final conclusions. Instead of employing it as a ‘rule-out’ test, the Microlife WatchBPO3 AFIB monitor is more appropriate in an opportunistic screening setting. Two studies have demonstrated a detection rate for newly diagnosed AF of up to 6.9% on a single reading [19, 20]. Since hypertension is the most independent risk factor for AF, the ability to simultaneously and accurately screen for PAF in patients who are referred for ambulatory BP monitoring is valuable. Given that the risk can be reduced with early and appropriate anticoagulant treatment, the identification of PAF is paramount [21, 22]. Even though a 24-h ECG may be the gold-standard detection tool, the analysis is cumbersome and time-consuming. Therefore, modified BP monitors are a more convenient and quicker alternative.

Considering that the sensitivity of the modified BP monitor to detect paroxysmal AF in the current study is 76.0%, there is a probability that 3 out of 10 patients with PAF could be overlooked by this screening tool. Our study reported an extremely low false-negative rate of 0.4%. On further investigation, the false-negative readings all stemmed from two patients that had a poor ambulatory BP recording of less than 65% successful readings, further emphasising that patients should achieve at least 80% valid BP readings for accurate detection of PAF. One plausible reason for false negative readings is how the pacemaker is arranged to protect the patient from atrial tachyarrhythmias, resulting in the modified BP monitor detecting the paced pulses delivered by the pacemaker, deeming the rhythm as regular, when in reality the patient is undergoing PAF. It is also possible that weak pulse signals as a result of an arrhythmia may go undetected by the modified BP monitor.

## Limitations

Due to the limited sample size, this study is not powered to produce statistically significant effects. Nevertheless, the simultaneous recording of the modified BP monitor and implanted pacemaker allows for accurate comparison. Other studies did not ensure that recordings were performed simultaneously [5, 16, 19, 20]. Compared to ambulatory ECG monitors, previous research has shown that pacemakers have a sensitivity of 98% and specificity of 100% to detect atrial tachyarrhythmias [23]. Despite this, employing a 24-h ECG monitor would have provided valuable information on any under-detection of arrhythmias (including those other than AF), short AHREs, or automatic mode switch events that may have caused either false negative or positive readings. Even though larger numbers of participants were enrolled in other studies, only a range of 1–3 readings was taken per individual. Contrarily, this study collected a total of 1595 readings over a 24-h period.

## Conclusion

In conclusion, the Microlife WatchBPO3 AFIB device has an acceptable diagnostic accuracy to detect PAF; however, movement artefact affects the accuracy of the readings. This modified BP monitor may potentially be useful as a screening tool for AF in patients at high risk of developing stroke.

## Electronic supplementary material

Below is the link to the electronic supplementary material.

Supplementary material 1 (TIFF 30 kb)
