# Supplementary figures and images for: Validation of the modified Microlife blood pressure monitor in patients with paroxysmal atrial fibrillation
Source: Clin Res Cardiol. 2019 Nov 7;109(7):802–9. doi: 10.1007/s00392-019-01567-y (PMC7308245; doi:10.1007/s00392-019-01567-y)

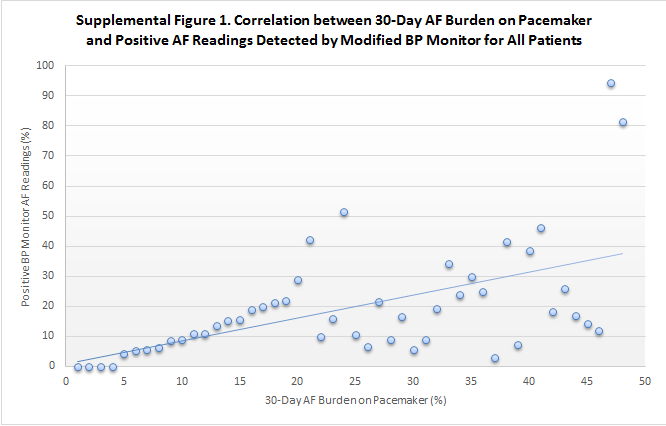

Supplement: Supplementary file 1 — Supplementary material 1 (TIFF 30 kb) [file 392_2019_1567_MOESM1_ESM.tif]
